# Intracranial hypertension: classification and patterns of evolution

**Published:** 2008-04-15

**Authors:** SM Iencean, AV Ciurea

**Affiliations:** * Neurosurgery, Hospital ‘Prof Dr N Oblu’, IasiRomania; ** University of Medicine and Pharmacy ‘Carol Davila’, Emergency Clinic HospitalRomania

**Keywords:** intracranial pressure, parenchymatous intracranial hypertension, vascular intracranial hypertension, cerebro–spinal fluid, idiopathic intracranial hypertension

## Abstract

Intracranial hypertension (ICH) was systematized in four categories according to its aetiology and pathogenic mechanisms: parenchymatous ICH with an intrinsic cerebral cause; vascular ICH, which has its aetiology in disorders of cerebral blood circulation; ICH caused by disorders of cerebro–spinal fluid dynamics and idiopathic ICH. The increase of intracranial pressure is the first to happen and then intracranial hypertension develops from this initial effect becoming symptomatic; it then acquires its individuality, surpassing the initial disease. The intracranial hypertension syndrome corresponds to the stage at which the increased intracranial pressure can be compensated and the acute form of intracranial hypertension is equivalent to a decompensated ICH syndrome.

The decompensation of intracranial hypertension is a condition of instability and appears when the normal intrinsic ratio of intracranial pressure – time fluctuation is changed. The essential conditions for decompensation of intracranial hypertension are: the speed of intracranial pressure increase over normal values, the highest value of abnormal intracranial pressure and the duration of high ICP values.

Medical objectives are preventing ICP from exceeding 20 mm Hg and maintaining a normal cerebral blood flow. The emergency therapy is the same for the acute form but each of the four forms of ICH has a specific therapy, according to the pathogenic mechanism and if possible to aetiology.

Intracranial hypertension, commonly abbreviated as ICH, is the elevation of intracranial pressure (ICP) due to the disturbance of regulatory intracranial pressure mechanisms. ICH is caused by changes in the volumes of brain parenchyma, cerebrospinal fluid and cerebral blood which exceed the compensating capacities of raised ICP [1, 12, 15]. The important increase in ICP causes the attainment of limiting conditions that induce clinical secondary alterations [7, 13, 16, 17].

## Classification of intracranial hypertension

Intracranial hypertension is classified in four forms beginning with aetiology, pathogenic mechanisms and patterns of increase in intracranial pressure (ICP):

### Parenchymatous intracranial hypertension

**Fig 1:**
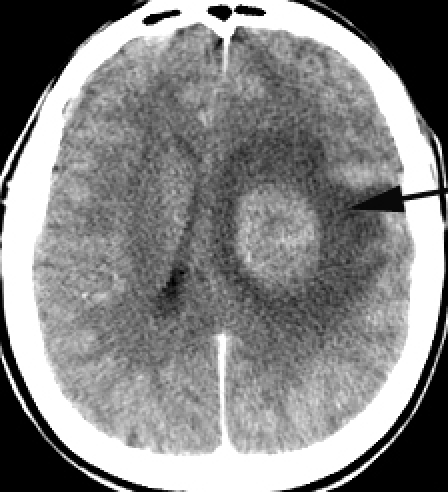
A contrast CT image of a deep frontal tumor with brain edema as a case of parenchymatous intracranial hypertension

 A primarily known brain aetiology causes alteration in intracranial volume then brain edema appears, evolving towards an increase in intracranial pressure. Parenchymatous ICH appears in expansive intracranial processes (tumors, haematomas, cerebral abscesses etc.), in traumatic brain edema, in general intoxication with neural toxins (exogenous or endogenous) etc. [2, 12, 20], (Fig 1).

The direct parenchymatous lesion occurs at first as a result of an intrinsic brain aetiology and of primary alterations in intracranial volume (expansive, compressive, hypoxic or traumatic brain edema). Frequently, the brain edema is sectorial and often there are differences between cerebrospinal compartments. There is a very rapid or a slow increase of ICP above 20 mmHg, but the duration of time of pathologic ICP is short because of decompensation. The parenchymatous ICH can have a complete evolution up to the acute form with brain stem ischemia or brain herniation [2, 11, 12, 25, 28].

### Vascular intracranial hypertension

 The development of brain edema and the increase of ICP are determined by disorders of cerebral blood volume (excluding the aetiology of parenchymatous ICH).

Brain edema occurs by ‘brain congestion’ following the increase of cerebral blood volume, caused by an important flow of blood or by a reduction or a stop in cerebral blood outflow. There is also a reduction of cerebro–spinal fluid (CSF) absorption involved in the decrease of cerebral blood flow.

Vascular ICH occurs in:

- Vascular cerebral diseases: cerebral venous thrombosis and superior sagittal sinus thrombosis, mastoiditis with transverse or sigmoid sinus thrombosis (the ‘otic hydrocephalus’ described by Simonds);
- Extracerebral diseases: hypertensive encephalopathies like acute hypertensive encephalopathy in cases of malignant hypertension of any cause, in glomerulonephritis, eclampsia etc., or in cerebral venous outflow reduction in congestive cardiac failure, superior vena cava syndrome or intrathoracic mass lesions.

**Fig 2:**
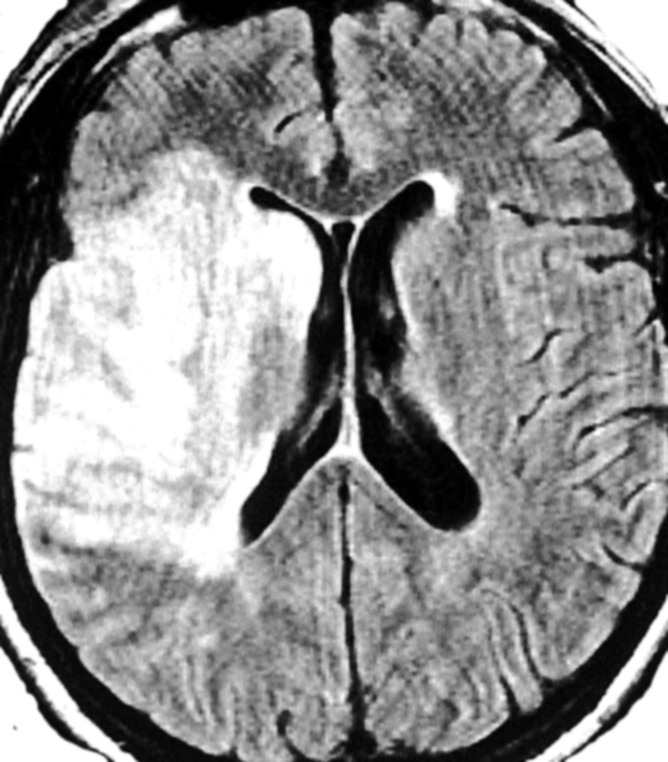
Middle cerebral right artery stroke: Subacute infarction of the right MCA territory, a type of vascular intracranial hypertension

The acute stoke is a cerebrovascular disease with different mechanisms but with two resultants on the brain: ischemia (85%) or haemorrhage (15%). The primary lesion in ischemic stroke can be an intra– or extracranial vascular disease. Brain edema and the increase of ICP occur through ultrafiltration after the increase of cerebral blood flow around infarction and/or a vasogenic brain edema (Fig 2). Usually there is an edema of the whole brain, but in some cases like in the ischemic stroke with ICH, there is a sectorial brain edema. Acute clinical presentations are due to the elevated ICP in vascular ICH but many symptoms are different depending on aetiology [3, 5, 8, 9, 15, 27].

Therefore, vascular aetiologies can individualize vascular types of intracranial hypertension:

- Cerebral venous thrombosis reduces venous outflow and determines low cerebrospinal fluid drainage and brain edema [3, 9, 21];
- Hypertensive encephalopathies cause brain swelling, both brain edema and congestive brain swelling with raised intracranial pressure (ICP) [11, 27];
- Ischemic strokes induce an increased capillary permeability with open brain-blood barrier, brain edema and severe elevation in ICP [15, 24].

These features of ICP increase depend on its aetiology: there is a low speed of ICP increase in cerebral venous thrombosis or a high speed in hypertensive encephalopathies or in ischaemic strokes. Also, the periods when ICP stays at high values are different depending on aetiologies of ICH: there is a long period in cerebral venous thrombosis and a short period in ischemic strokes.

### Intracranial hypertension caused by disorders of cerebro–spinal fluid dynamics (CSF)

 The cerebro–spinal fluid dynamics include all aspects of CSF circulation from production to absorption [6, 11, 18].

**Fig 3:**
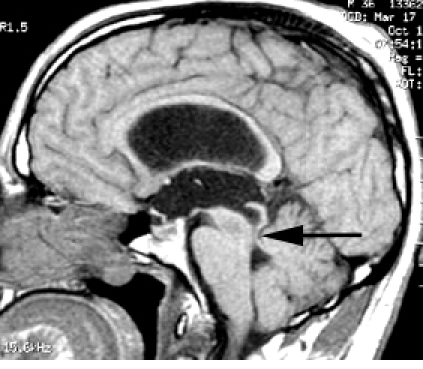
MR image demonstrates an aqueductal stenosis, like an intracranial hypertension determined by disorders of cerebro–spinal fluid circulation

The disorders of the cerebrospinal fluid dynamics are:

Disorders of CSF circulation. They result from obstruction of the pathways of CSF circulation. Tumors, haemorrhages, aqueductal stenosis and infections can cause obstruction at either point of the pathways. Clinical features are similar to an obstructive hydrocephalus; CSF accumulates within ventricles because of CSF flow blockage [5, 19, 23] (Fig 3).

Disorders of CSF absorption. The apparent mechanism is the occlusion of arachnoid villi, perhaps by blood and inflammatory mediators. This form includes the cases of reduction of cerebrospinal fluid absorption in acute meningitis, in subarachnoid haemorrhage, carcinomatous meningitis, and chronic meningitis – sarcoidosis. There is a thickening of the leptomeninges and an involvement of arachnoid granulations with a drop of CSF absorption and an acute ‘communicating’ hydrocephalus with acute ICH syndrome [6, 22, 23].

### Idiopathic intracranial hypertension: its aetiology can not be established

Idiopathic intracranial hypertension (IdIH) is the persistent increase in intracranial pressure in the absence of any intracranial lesions: intracranial tumor, hydrocephalus, intracranial infections, dural sinus thrombosis or hypertensive encephalopathy [14, 15, 26]. Idiopathic intracranial hypertension corresponds partially to the old term of pseudo tumor cerebri; in 1969 Buchheit introduced the term idiopathic intracranial hypertension, replacing the term ‘benign intracranial hypertension’, abandoned due to visual disturbances. IdICH occurs in endocrine, metabolic and haematological diseases, hormonal treatments etc. The frequency of ICH syndrome is the lowest in regard to the diseases to which it is correlated. The IdIH diagnosis is made only after measuring intracranial pressure and full neuroimaging exploration.

Diagnostic criteria for idiH are [5, 7, 14, 20]:

- CSF pressure is greater than 25 cm H_2_O, [14, 15]
- Normal CSF
- Symptoms of increased intracranial pressure: papilledema, headache, no signs of neurological localisation.
- CT scanning or magnetic resonance imaging show a normal cranial–cerebral state, without no clinical or neuroimaging suspicion of venous intracranial thrombosis

Females are predominant, with female/male ratio being as high as 8:1; the maximum incidence occurs in the third decade. In young obese women, the incidence of IIH has been shown to reach 20 cases per 100,000.

The increase of ICP is gradual. This allows a good compensation of the raised ICP. The increase of ICP is very important, up to 60–80 mmHg but the brain vascular auto regulation compensates the increase in ICP and maintains cerebral blood flow.

There are some theories for the apparition of idiopathic ICH (IdIH): a) increased resistance to CSF absorption; b) increased CSF production; and c) increased venous sinus pressure (some authors do not exclude IdIH from occurring because of a dural sinus thrombosis) [4, 8, 19, 26].

The increased resistance to CSF absorption with reduced resorption changes the CSF chemical composition and excludes IdIH. The increase in sagittal sinus venous pressure causes an increase in intracranial pressure, but the pathogenesis is vascular and vascular intracranial hypertension occurs.

Other hypothesis is presented further on:

The anatomical and physiological data show that the mechanisms of interstitial fluid formation at the blood–brain barrier (BBB) and of cerebrospinal fluid (CSF) at the choroid plexus are very similar. In IdIH, many pathological conditions can induce simultaneous hypersecretion of CSF and of brain interstitial fluid therefore, the high intracranial pressure (ICP) of CSF will be equalized simultaneously by that of the brain’s interstitial fluid. The cerebral blood flow is maintained quasinormal in IdIH, even increased, so that a fast absorption of CSF and of interstitial fluid occurs and the brain injury is insignificant despite high intracranial pressure [6, 14, 15].

Therefore, idiopathic intracranial hypertension can occur through the simultaneous hypersecretion of the cerebrospinal fluid and of cerebral interstitial fluid followed by a rapid circulation and absorption of these fluids based on a fast cerebral blood flow. The edema is in the entire brain and there is no difference of raised ICP between cerebrospinal compartments. The duration of pathological values of ICP is prolonged and symptoms are reduced: headache, papilledema and visual loss, early or late. Therefore IdIH evolves only to an incomplete ICH syndrome.

Parenchymatous ICH evolves to the acute form by brain stem ischemia or brain herniation because of ICP difference between the three cerebrospinal compartments; vascular ICH and ICH in disorders of CSF dynamics evolve usually including the ICH syndrome. The idiopathic ICH is only an incomplete ICH syndrome.

The main features of these four forms of ICH are presented in Table 1

**Table 1:** Classification of intracranial hypertension based on pathogenesis

Parenchymatous	Vascular	CH caused by disorders of CSF dynamics	CH caused by disorders of CSF dynamics
ICH	ICH	CSF obstruction	Disorders of CSF absorbtion	Idiopathic ICH
Clear aetiology: brain lesion or injury	Aetiology: brain or general vascular injury	Clear aetiology: CSF obstruction	Aetiology: meningitis etc.	Unknown aetiology or various unspecified aetiology named ‘associated factors’
Perifocal edema or sectorial brain edema	Generalized or sectorial brain edema	Obstructive hydrocephalus and hydrocephalic brain edema	Non–obstructive hydrocephalus and generalized brain edema	Generalized brain edema, in equilibrium intraventricular pressure
High speed of ICP increase	Medium speed of ICP increase	High speed of ICP increase	Medium speed of ICP increase	Very slow speed of ICP increase
Critical threshold of ICP ~ 20 mm Hg	Critical threshold of ICP ~ 20 mm Hg	Critical threshold of ICP ~ 20 mm Hg	Critical threshold of ICP ~ 20 mm Hg	High ICP ~ 60 – 80 mm Hg
ICP difference of cerebrospinal compartments	Usually no ICP difference between cerebrospinal compartments	ICP difference of cerebrospinal compartments	No ICP difference between cerebrospinal compartments	Raised ICP is constant in cerebrospinal compartments
ICP decreases the CBF auto regulation	Vascular injury diminishes CBF auto regulation	ICP decreases the CBF auto regulation	Inflammatory vasculitis can disturbs CBF auto regulation	High ICP do not decreases the CBF auto regulation
Short period of highest ICP action	Extended period of pathologic ICP values	Short period of highest ICP action	Various period of pathologic ICP	Very prolonged high ICP action
Complete evolution to decompensated ICH : brain herniation, brain stem ischemia	Evolution varying with aetiology, usually complete ICH syndrome	Complete evolution to decompensated ICH	Evolution depending on aetiology; usually complete or incomplete ICH syndrome	Evolution : incomplete ICH syndrome,possible blindness Discordance: apparently satisfactory clinicalcondition/high ICP and papillary edema
Aetiological treatment: often neurosurgical	Symptomatic and aetiological treatment	Neurosurgical treatment : etiologic or shunt	Aetiological and/or symptomatic treatment; shunt	Therapy for possible causal or ‘associated factors’; lumbar–peritoneal shunt,optic nerve decompression

## The course of intracranial hypertension

Increased intracranial pressure is initially an alarm signal for intracranial hypertension; later on, the increase in intracranial pressure is accompanied by symptoms that represent the ICH syndrome and becomes a pathogenic mechanism in itself making intracranial hypertension appear as an acute disease with individual evolution [13]. The pressure–time fluctuation defines the rise of ICP relying on the duration of time of high ICP that induces reduction of the auto regulation period of cerebral blood circulation. This pressure–time fluctuation depends on the speed of ICP increase and there are more than one main patterns of ICP increase corresponding to the types of intracranial hypertension. The maximum instability conditions correspond to the decompensation that occurs through brain stem ischemia or brain herniation.

The types of brain diseases and the forms of ICH have a characteristic ICP increase and ICP monitoring shows some features [13, 15]:

- the speed of ICP elevation to the normal value of 20 mmHg and over this normal limit
- the peak value of increased ICP
- the period of pathological values of ICP
- the length of recurrence to normal values of ICP
- the frequency of occurrence of ICP increase

In acute situations of parenchymatous intracranial hypertension like haematomas or traumatic brain edema, when a new volume develops very quickly into the cranium, there is a very rapid increase of ICP to 20 mm Hg and then over this value. The decompensation if ICH is rapid due to the high speed of the new volume development and the exceeding of compensatory capacities.

In gradual evolution of the parenchymatous intracranial hypertension, as in brain tumors, cerebral abscesses etc. when the new volume develops slowly into the cranium, there is a slow increase of ICP to highest normal values and then ICP rises rapidly over normal. The subclinical period and the period of ICH syndrome are longer like in acute cases, but the decompensation of ICH is rapid because of the exceeding of compensatory capacities.

In some vascular ICH determined by hypertensive encephalopathies, the rapidity of ICP increase till the normal limit is medium as a rule and the period of high ICP values is long. 

The reduction of cerebrospinal fluid absorption in meningitis, subarachnoid haemorrhage and in the cases of cerebral venous thrombosis, superior sagittal sinus thrombosis and usually in cerebral vascular disorders has a slow increase of ICP to and over the normal limit.

The gradual increase of ICP in IdIH allows a good compensation and a quasi–normal cerebral blood flow. There is a very slow ICP increase and the pathological values of ICP can be very high, up to 60–80 mmHg, only with an incomplete ICH syndrome very prolonged and without decompensation.

## Conclusions

- The main features that determine the clinical course of ICH are: speed of ICP increase to and over the normal limit, critical thresholds and the highest value of ICP, period of pathological value of ICP, length of recurrence to normal value and the frequency of occurrence of ICP increase.
- Pressure–time fluctuation is the dynamic element in the progression and decompensation of intracranial hypertension. The evolution of ICH is through exceeding the critical thresholds of the ICP equivalent to each stage and decompensation corresponds to the acute critical pressure–time fluctuation.
- The intracranial hypertension syndrome corresponds to the stage at which increases in ICP can be compensated, equivalent to the chronic form of ICH, while ICH disease is the acute form and represents a decompensated ICH syndrome.
- The classification is based on aetiopathogenesis of ICH:
  - Parenchymatous intracranial hypertension with an intrinsic cerebral cause; it can reach the acute form;
  - Vascular intracranial hypertension's aetiology lies in disorders of cerebral blood circulation and advances usually to an ICH syndrome;
  - Intracranial hypertension determined by disorders of cerebro–spinal fluid dynamics;
  - Idiopathic intracranial hypertension without aetiology (which is an incomplete ICH syndrome).

## References

[1] Allen CH, Ward JD (1998). An evidence based approach to management of increased intracranial pressure. Critical Care Clin.

[2] Betz AL, Iannotti F, Hoff JT (1989). Brain edema: a classification based on blood-brain barrier integrity. Cerebrovasc Brain Metab Rev.

[3] Bateman GA (2008). Arterial inflow and venous outflow in idiopathic intracranial hypertension associated with venous outflow stenoses. J Clin Neurosci.

[4] Biousse V, Ameri A, Bousser MG (1999). Isolated intracranial hypertension as the only sign of cerebral venous thrombosis. Neurology.

[5] Biousse V, Tong F, Newman NJ (2003). Cerebral Venous Thrombosis. Curr Treat Options Cardiovasc Med.

[6] Borgesen SE, Gjerris F (1987). Relationship between intracranial pressure, ventricular size and resistance to CSF outflow. J.Neurosurg.

[7] Donovan JP (1998). Cerebral oedema and increased intracranial pressure in chronic liver disease. Lancet.

[8] Farb RI, Vanek I, Scott JN, Mikulis DJ, Willinksy RA, Tomlinson G, TerBrugge KG (2003). Idiopathic intracranial hypertension: The prevalence and morphology of sinovenous stenosis. Neurology.

[9] Ferro JM, Canhao P (2008). Acute treatment of cerebral venous and dural sinus thrombosis. Curr Treat Options Neurol.

[10] Friedman DI, Streeten DH (1998). Idiopathic intracranial hypertension and orthostatic edema may share a common pathogenesis. Neurology.

[11] Gronbaek E (2002). Acute and chronic hypertensive headache and hypertensive encephalopathy. Cephalalgia.

[12] Iencean SM (2003). Brain edema–a new classification. Med Hypotheses.

[13] Iencean SM (2002). A new classification and a synergetical pattern in intracranial hypertension. Med Hypotheses.

[14] Iencean SM (2006). Idiopathic intracranial hypertension . Review and hypothesis of the pathogenesis. Res J Med and Medical Sciences.

[15] Iencean SM (2006). Hipertensiunea intracraniana.

[16] Kleinschmidt JJ, Digre KB, Hanover R (2000). Idiopathic intracranial hypertension.Relation to depression,anxiety and quality of life. Neurology.

[17] Larsen GY, Goldstein B (1999). Increased intracranial pressure. Pediatrics in review.

[18] Mariak Z (2000). Intracranial pressure processing with artificial Neural Networks: classification os signal properties. Acta Neurochir.

[19] Mihorat TH (1992). Classification of the cerebral edemas with reference to hydrocephalus and pseudotumor cerebri. Childs Nerv Syst.

[20] Moraine JJ, Berre J, Melot C (2000). Is cerebral perfusion pressure a major determinant of cerebral blood flow during head elevation in comatose patients with severe intracranial lesions?. J.Neurosurg.

[21] Owler BK (2003). Pseudotumor cerebri syndrome: venous sinus obstruction and its treatment with stent placement. J.Neurosurg.

[22] Pukkila–Worley R, Mylonakis E (2008). Epidemiology and management of cryptococcal meningitis: developments and challenges. Expert Opin Pharmacother.

[23] Salman MS (1997). Benign intracranial hypertension or communicating hydrocephalus: factors in pathogenesis. Med Hyp.

[24] Schneck MJ (1998). Treating elevated intracranial pressure : Do we raise or lower the blood pressure?. Crit Care Med.

[25] Stocchetti N (1999). Intracranial hypertension in head injury : management and results. Int Med Care.

[26] Stevens SA, Stimpson J (2008). A model for idiopathic intracranial hypertension and associated pathological ICP wave–forms.. IEEE Trans Biomed Eng.

[27] Thambisetty M, Biousse V (2003). Hypertensive brainstem encephalopathy: clinical and radiographic features. J Neurol Sci.

[28] Timofeev I, Czosnyka M (2008). Effect of decompressive craniectomy on intracranial pressure and cerebrospinal compensation following traumatic brain injury. J Neurosurg.

